# The acceptability of vaginal smear self-collection for screening for cervical cancer: a systematic review

**DOI:** 10.6061/clinics/2017(03)09

**Published:** 2017-03

**Authors:** Natalia Serrano Doratioto Faria Braz, Noely Paula Cristina Lorenzi, Isabel Cristina Esposito Sorpreso, Lana Maria de Aguiar, Edmund Chada Baracat, José Maria Soares

**Affiliations:** Hospital das Clínicas da Faculdade de Medicina da Universidade de São Paulo, Disciplina de Ginecologia do Departamento de Obstetrícia e Ginecologia, São Paulo/SP, Brazil

**Keywords:** Cervical Cancer, Vaginal Smear Self-Collection, Cancer Screening, Method Acceptance, Systematic Review

## Abstract

Cervical cancer is a major cause of death in adult women. However, many women do not undergo cervical cancer screening for the following reasons: fear, shame, physical limitations, cultural or religious considerations and lack of access to health care services. Self-collected vaginal smears maybe an alternative means of including more women in cervical cancer screening programs. The objective of this systematic review was to evaluate the acceptability of vaginal smear self-collection for cervical cancer screening. We selected articles from PubMed, the Cochrane Library and Embase that were published between January 1995 and April 2016. Studies written in English, French, Italian, Portuguese or Spanish that involved women between 18 and 69 years of age who had engaged in sexual intercourse were included in this review. The review was performed in accordance with the PRISMA (Preferred Reporting Items for Systematic Reviews and Meta-Analyses) statement. Nineteen studies were ultimately evaluated in this review. Most of the included studies (n=17) demonstrated that the self-collection method exhibited outstanding acceptability among women with respect to cervical cancer screening, and only two studies indicated that self-collection exhibited low acceptability among women in this context. The acceptability of self-collection was determined subjectively (without standardized questionnaires) in 10 studies (53%) and via structured and validated questionnaires in the remaining studies. The results of our review suggest that the self-collection method is well-accepted and may therefore encourage greater participation in cervical cancer screening programs. However, additional studies are required to verify these results.

## INTRODUCTION

Screening for cancer of the cervix has intensified in recent decades, enabling the identification of precursor lesions and cancer at earlier disease stages, thereby increasing patient survival. However, many patients still die from this disease 1, which is the most common cancer affecting women after non-melanoma skin cancer, breast cancer and colorectal cancer 1. Cervical cancer is also the fourth-leading cause of malignancy-related death among women in Brazil 2. Therefore, additional means of screening for this disease, which remains a great public health concern, are needed.

The collection of cervix-vaginal cytology samples by health care professionals is generally an effective tool for performing cervical cancer screening. However, many women do not undergo this test for the following reasons: fear, embarrassment, functional or physical limitations, cultural or religious reasons and even lack of access to health services 3,4. In general, women living in rural areas or on the outskirts of large cities have lower education levels and are of lower social and economic statuses than their counterparts in urban areas. Additionally, these women are more likely to have their first sexual intercourse prematurely and often have more sexual partners and more children than other women. Consequently, clinicians have less opportunities to implement preventative health measures among these populations 5.

The vaginal smear self-collection method was created to provide women with access to cervical cancer screening, as patients can perform smear collections themselves and then forward their smears to the appropriate facility for further analysis 5-7. Thus, this method has the potential to increase participation in cervical cancer screening and to facilitate the incorporation of populations living on the outskirts of major centers, including prisoners, into screening programs. However, cultural and psychological factors (fear or fear of self-manipulation) may limit the effectiveness of this method. Our systematic review aimed to assess the acceptability of the self-collection method among women.

## METHODS

We conducted a systematic review of studies regarding the acceptability of using the vaginal self-collection method for cervical cancer screening among women. This study was conducted in accordance with the recommendations established by PRISMA (Preferred Reporting Items for Systematic Reviews and Meta-Analyses) 8.

We consulted Medline, the Cochrane Library and Embase to identify relevant studies published from January 1995 (first report) to April 2016. We did not impose any restrictions regarding publication dates. We searched for texts published in English, French, Italian, Spanish and Portuguese. We used search keywords that were in accordance with our selected P.I.C.O. ("patient", "intervention", "control" and "outcome"), and the specific search strategies utilized for electronic databases are summarized in Figure 1. The process of manuscript retrieval is described in Figure 2. Publications listed in the references sections of retrieved articles were also retrieved. Studies involving women over 18 years of age who had engaged in sexual intercourse (P) and submitted to the self-collection of vaginal smears (I) were included in this review. The control group was conventional smears (C). The outcome was the acceptability of the self-collection of vaginal smears for assessing uterine neoplasm in the cervix (O). Retrospective studies or studies for which we did not have access to the full text were excluded. The study selection process and the evaluations of the titles and abstracts obtained through the above searches were conducted in an unbiased manner in strict accordance with the inclusion and exclusion criteria of this study by two researchers (NDSFB and NPCL) skilled in the preparation of systematic reviews. The original articles were subsequently critically evaluated to decide whether they should be included in the review. Meta-analysis was not performed due to variability regarding the methods used for assessing self-collection method acceptability. A third reviewer (JMSJ) was consulted when there was disagreement regarding the selection of studies among the researchers. The information obtained from the selected studies was entered into a table including information regarding the names of the authors, the years of publication, the study designs, the numbers of patients, the ages of the patients, the index test (self-collection) and the reference test (conventional collection).

## RESULTS

A total of 290 studies were initially retrieved; 267 of these studies were excluded by applying the aforementioned exclusion criteria. After the references of the selected articles were cross-checked, we included another manuscript. After reading and analyzing articles, we excluded four manuscripts for being in a language that does not satisfy the inclusion criteria. We excluded additional manuscripts because the studies described did not provide detailed results. Thus, 19 manuscripts were ultimately included in this review. The information obtained from the selected studies was entered into a table including information on author names, the publication year, country, study design, the numbers of participants, the ages of the patients, acceptability and the method used (Table 1).

A total of 18,202 participants were included in this study 9-27. Only two studies (10.5%) demonstrated that the self-collection method exhibited low acceptability among women 9,23. The acceptability of the self-collection method among women was determined subjectively (without standardized questionnaires) in ten studies (52.6%) 10,13-16,18,20-21,23,26 and via structured and valid questionnaires in the remaining studies. Only five studies were randomized. These results are summarized in Table 1.

The women enrolled in the two studies demonstrating that the self-collection method exhibited low acceptability preferred to continue undergoing screenings performed by health care professionals because they were afraid of not performing the sampling properly or were concerned about experiencing some discomfort during the procedure 9,23. Additionally, some women questioned the validity of self-collected smear results and wondered about the possibility of medical appointments being replaced with self-collection procedures 23. The participants enrolled in these studies, particularly women over 50 years of age, also reported that the explanations regarding how to perform self-collection were confusing and inadequate 9,23.

Among the 17 manuscripts demonstrating that the self-collection method exhibited high acceptability among women, nine performed only subjective evaluations 10,13-16,18,20-21,26, whereas the remaining eight studies used standardized and validated questionnaires.

The following main points were addressed across these questionnaires: 1) the psychosocial aspects of self-collection, such as shame; 2) the feasibility of self-collection, such as perormance reliability; 3) the practicality of self-collection; 4) the desire to perform self-collection again 11-12; 5) characteristics related to life style and reproductive considerations, which were determined using self-administered questionnaires 11,17; 6) the acceptability of self-collection compared with traditional sample collection 17,19,22; 7) the likelihood of recommending self-collection to a family member or friend 24,25-27; 8) the grade of discomfort associated with self-collection, determined using a 5-point Likert scale 27; 9) participants’ knowledge regarding HPV and cervical cancer 11-12; 10) media handling, which was addressed using simple questions, such as "Was the procedure uncomfortable?" or ”Were you embarrassed?" 17,24; and 11) participants’ assessments of the instructions that they were provided 12,22. Thus, the studies were heterogeneous with respect to the information collected by the different types of questionnaires used therein. However, most of the studies indicated that the self-collection method possessed the following advantages over the conventional screening method: easier and faster implementation and lower costs 9-15, 22-25. Prior detailed explanations regarding the method played a fundamental role in the opinions of the participants regarding the method and their acceptance of the method 9,23.

## DISCUSSION

Cervical cancer remains a public health challenge 1-5. The results of this review indicate that vaginal smear selfcollection is a well-accepted method that may increase participation in cervical cancer screening 10-22,24-27. However, no standardized questionnaire for evaluating the acceptability of this method exists 10-22,24-27, and better explanations regarding the performance of this method are necessary to improve patient participation in cancer screening 9,23.

Low acceptability of the method among women, which was noted in two studies, was mainly attributed to participant insecurity regarding appropriate sample handling 9,23. The women enrolled in these two studies reported having difficulty understanding the tested approach due to a lack of knowledge regarding their own bodies (their anatomy) 9,23. Participants also expressed concern regarding the possibility that medical appointments could be replaced by vaginal smear self-collection procedures 23. These findings indicate that health education is important with respect to the acceptance of new technologies and treatments 28-29.

The studies demonstrating that the self-collection method exhibited high acceptability among women noted that the ease and rapidity of the self-collection method provide women with greater autonomy with respect to collecting vaginal material, thereby increasing participation in screening programs and complementing the classical methods utilized by health care professionals 21-22,24-27, particularly among populations with difficulty accessing health care facilities, to ultimately facilitate increases in the rate of early cervical cancer diagnosis. The review also noted that the guidelines and explanations pertaining to the performance of the procedure played an important role in increasing patient confidence in and acceptance of the method 9,23. Therefore, the introduction of self-collection should be preceded by community education regarding both the method and the female genitourinary tract.

Several studies used subjective questions regarding the self-collection method, whereas others used complex questionnaires encompassing questions regarding the psychological impact of the self-collection method 9,23, making it difficult to compare the studies, a weakness of this analysis. In addition, the numbers of participants involved in the included studies varied, ranging from <30 participants to >1,000 participants 9-27. Moreover, only five studies included in this review were randomized, indicating that additional randomized studies that feature long follow-up periods and include participants who have received prior education regarding the self-collection method are necessary.

The findings of this systematic review indicate that vaginal smear self-collection is a well-accepted method that may increase participation in cervical cancer screening. However, barriers exist with respect to the use of the self-collection procedure among women who are uncomfortable performing the procedure or uncertain regarding the validity of its results. Thus, additional randomized, prospective and long-term follow-up studies regarding the acceptability of the vaginal smear method are needed.

## AUTHOR CONTRIBUTIONS

Braz NS was responsible for the design, revision, analysis and manuscript writing. Lorenzi NP was responsible for the design, revision, analysis and manuscript writing. Sorpreso IC was responsible for the design and revision of the manuscript. Aguiar LM was responsible for the design and revision of the manuscript. Baracat EC was responsible for the design, revision, analysis and manuscript writing. Soares-Júnior JM was responsible for the design, revision, analysis and manuscript writing.

## Figures and Tables

**Figure:**
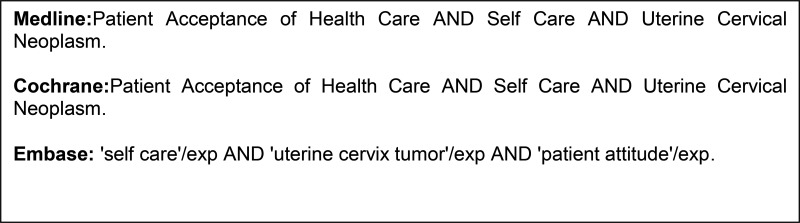
Databases and search strategies.

**Figure:**
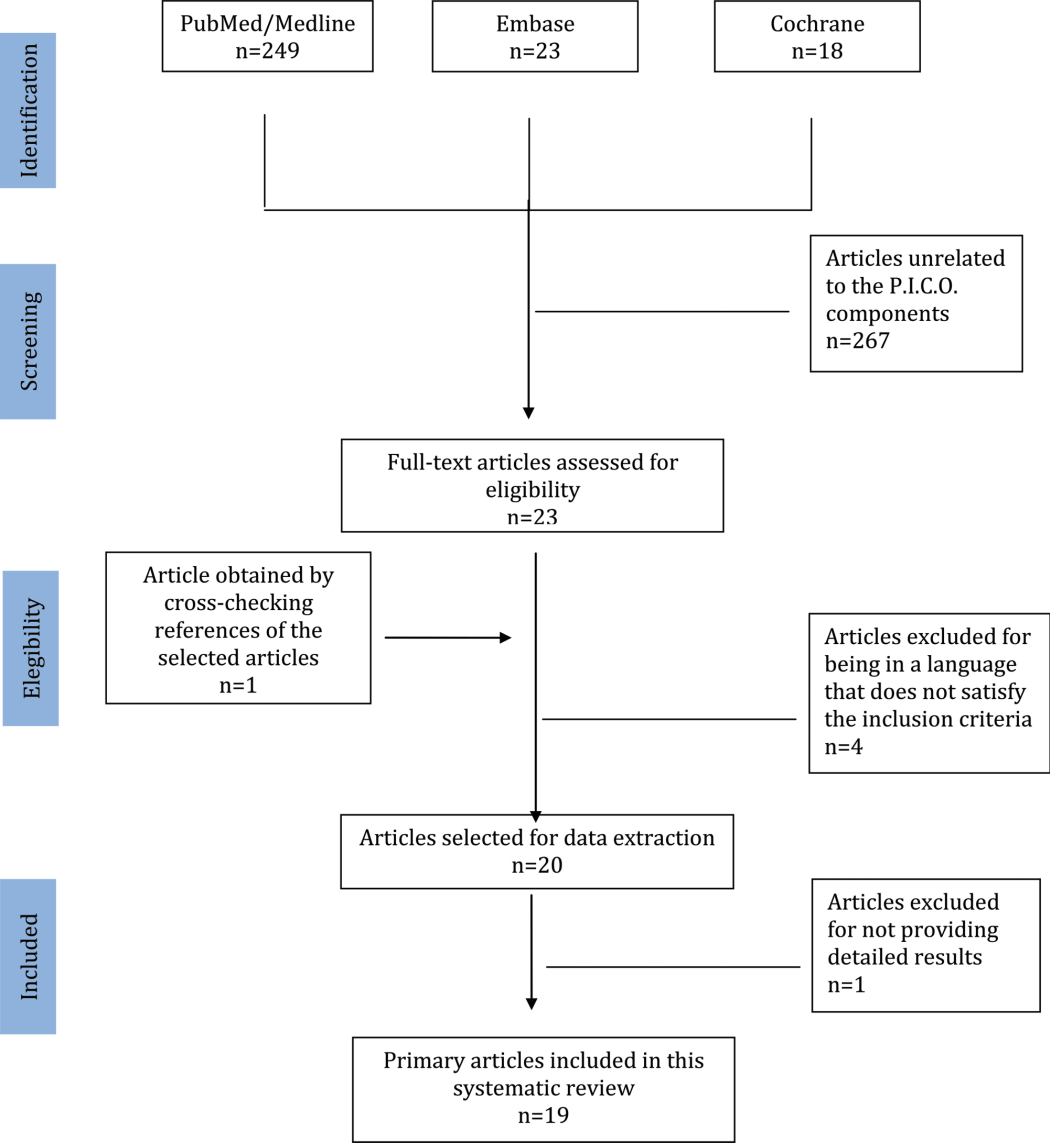
The algorithm used for this systematic review.

**Table 1:** Studies included in the systematic review.

	Authors and publication year	Country	Age (years)	Type	Number of participants	Acceptability	Method used
1)	Szarewski et al. (9)	United Kingdom	21 – 65	Transversal	28	Low	Questionnaire
2)	Mitchell et al. (10)	Uganda	30 – 65	Transversal	300	High	Subjective
3)	Szarewski et al. (11)	United Kingdom	29 – 65	Randomized	3000	High	Questionnaire
4)	Ortiz et al. (12)	USA	18 – 34	Case-control	100	High	Questionnaire
5)	Cerigo et al. (13)	Canada	18 – 69	Case-control	93	High	Subjective
6)	Quincy et al. (14)	Nicaragua	25 – 60	Case-control	250	High	Subjective
7)	Fielder et al. (15)	USA	18 – 69	Randomized	483	High	Subjective
8)	Penaranda et al. (16)	Mexico	30 – 65	Transversal	21	High	Subjective
9)	Sultana et al. (17)	Australia	30 – 69	Randomized	8000	High	Questionnaire
10)	Vanderpool et al. (18)	USA	30 – 64	Transversal	31	High	Subjective
11)	Racey et al. (19)	Canada	30 – 70	Randomized	818	High	Questionnaire
12)	Penaranda et al. (20)	Mexico	30 – 65	Transversal	110	High	Subjective
13)	Sultana et al. (21)	Australia	30 – 69	Transversal	35	High	Subjective
14)	Crofts et al. (22)	Cameroon	30 – 65	Transversal	450	High	Questionnaire
15)	Fargnoli et al. (23)	Switzerland	24 – 67	Transversal	125	Low	Subjective
16)	Sultana et al. (24)	Australia	30 – 69	Transversal	1521	High	Questionnaire
17)	Dareng et al. (25)	Nigeria	18 – 69	Transversal	600	High	Questionnaire
18)	Boggan et al. (26)	Haiti	25 – 65	Case-control	1845	High	Subjective
19)	Wong et al. (27)	China	35 – 65	Randomized	392	High	Questionnaire
